# Composition, Physicomechanical, and Clinical Behaviour of Nanohybrid Resin Composites: A Narrative Review

**DOI:** 10.3390/jfb17100513

**Published:** 2026-10-12

**Authors:** Alexia-Ecaterina Cârstea, Ioana-Irina Anton, Lucian-Toma Ciocan, Vlad-Gabriel Vasilescu, Marina Imre, Andreea-Cristiana Didilescu, Silviu-Mirel Pițuru, João Malta Barbosa, Roxana-Romanița Ilici, Bogdan Dimitriu, Ana-Maria Cristina Țâncu

**Affiliations:** 1 Discipline of Dental Prosthesis Technology, Faculty of Dentistry, “Carol Davila” University of Medicine and Pharmacy, Dionisie Lupu Street, No. 37, District 2, 020021 Bucharest, Romania; alexia-ecaterina.carstea2023@stud.umfcd.ro (A.-E.C.); ioana-irina.anton2023@stud.umfcd.ro (I.-I.A.); 2 Discipline of Prosthodontics, Faculty of Dentistry, “Carol Davila” University of Medicine and Pharmacy, 37 Dionisie Lupu Street, District 2, 020021 Bucharest, Romania; marina.imre@umfcd.ro (M.I.); anamaria.tancu@umfcd.ro (A.-M.C.Ț.); 3 Department of Embriology and Microbiology, Faculty of Dentistry, “Carol Davila” University of Medicine and Pharmacy, 37 Dionisie Lupu Street, District 2, 020021 Bucharest, Romania; andreea.didilescu@umfcd.ro; 4 Department of Organization, Professional Legislation and Management of the Dental Office, Faculty of Dental Medicine, “Carol Davila” University of Medicine and Pharmacy, 17-23 Plevnei Str., 020021 Bucharest, Romania; silviu.pituru@umfcd.ro; 5 Department of General Dentistry and Comprehensive Care in Operative & Digital Dentistry, New York University College of Dentistry, 386 Park Avenue, 10th Floor, New York, NY 10016, USA; jmbarbosa@nyu.edu; 6 Department of Oral Rehabilitation, Egas Moniz School of Health & Science, Campus Universitário, Quinta da Granja, Monte de Caparica, 2829-511 Caparica, Portugal; 7 Department of Prosthodontics, Lisbon University College of Dental Medicine, Rua Professora Teresa Ambrósio, Cidade Universitária, 1600-277 Lisbon, Portugal; 8 EV Oral Center, Rua Dom Manuel da Conceição Santos, 59A, 7005-451 Évora, Portugal; 9 Teeth and Dental Arches Morphology and Dental Materials Department, Faculty of Dentistry, “Carol Davila” University of Medicine and Pharmacy, 17-21 Calea Plevnei Street, Sector 1, 010221 Bucharest, Romania; roxana.ilici@umfcd.ro; 10 Department of Endodontics, Faculty of Dental Medicine, “Carol Davila” University of Medicine and Pharmacy, 8 Eroilor Sanitari Blvd, 050474 Bucharest, Romania; bogdan.dimitriu@umfcd.ro

**Keywords:** composite resins, dental materials, materials testing, hardness, color, surface properties, dental restoration, permanent, nanohybrid composite

## Abstract

Nanohybrid resin composites are the dominant class of direct restorative materials, yet evidence on individual products is dispersed and difficult to compare. This narrative review appraises the composition, physicomechanical, chemical, optical, and clinical behaviour of Filtek™ Z550 (introduced in 2012), an extensively tested nanohybrid, and places its profile in the context of its class. Methods: A structured search of PubMed and the Cochrane Library with manual reference screening was performed; no formal risk-of-bias or certainty-of-evidence assessment was undertaken. Original in vitro and clinical studies were screened against predefined PICO-oriented criteria and synthesised qualitatively. Results: Twenty-seven studies were included, twenty-six in vitro and one clinical. The material was consistently described as a multicomponent dimethacrylate matrix reinforced with a bimodal zirconia and silica filler system at approximately 82 percent by weight, a configuration linked to high early flexural strength, elevated surface hardness, and favourable polishability. Artificial ageing reduced microhardness by roughly 16 to 24 percent and increased wear, three-point flexural strength fell below the ISO 4049 minimum after thirty days of ageing in artificial saliva, and low-pH and oxidative challenges degraded gloss and colour. Clinical data were limited to a single eighteen-month trial reporting high retention with more frequent transient postoperative sensitivity. Conclusions: The laboratory evidence indicates favourable early physicomechanical and esthetic behaviour that is sensitive to hydrolytic ageing and to polymerisation quality; clinical evidence rests on one short-term trial, so statements about clinical suitability are preliminary.

## 1. Introduction

Resin-based composites have progressed from esthetically limited, mechanically fragile formulations into the principal materials used for direct tooth-coloured restorations in both anterior and posterior regions [1]. This progress was driven largely by developments in filler technology, since the size, morphology, and volume fraction of the inorganic phase govern the strength, wear resistance, and surface behaviour that clinical service demands [2]. Contemporary materials are commonly grouped by their filler architecture into microfilled, hybrid, microhybrid, nanofilled, and nanohybrid categories, alongside handling-defined variants such as flowable, packable, and bulk-fill composites [3]. Packable composites were developed with coarse or high-fraction fillers to improve condensability in posterior cavities, whereas nanofilled and nanohybrid composites combine discrete nanoparticles with nanoscale clusters or conventional glass fillers to reconcile mechanical reinforcement with a fine, polishable surface [1]. Nanohybrid composites now represent the dominant category for universal restorative use [4].

The rationale for reducing filler dimensions rests on the physics of both reinforcement and light interaction. Very fine particles increase the interfacial area available for stress transfer, and when their size falls well below the wavelength of visible light, they limit scattering and preserve translucency [5]. Particle size distribution also sets the maximum packing fraction, because small particles occupy the interstices between larger ones and raise the attainable filler content without an unworkable increase in viscosity [2]. Across commercial nanohybrids, differences in filler fraction, particle geometry, and silanisation account for much of the between-product variation in hardness, modulus, and wear [2]. Well-characterised examples of the class include Tetric EvoCeram, GrandioSO, Ceram.X, IPS Empress Direct, Evetric, and Filtek Z550, which differ in filler loading and monomer blend while sharing the bimodal nano-plus-cluster design [3].

The organic phase of these materials is typically built from a blend of dimethacrylate monomers, most often bisphenol-A glycidyl dimethacrylate (Bis-GMA), urethane dimethacrylate (UDMA), bisphenol-A ethoxylated dimethacrylate (Bis-EMA), triethylene glycol dimethacrylate (TEGDMA), and poly(ethylene glycol) dimethacrylate (PEGDMA) [6]. Each monomer contributes a distinct balance of viscosity, reactivity, and network rigidity, so the proportions selected by a manufacturer influence the degree of conversion, the mechanical modulus, and the hydrophilicity of the cured polymer [7]. High-molecular-weight monomers such as Bis-GMA and Bis-EMA increase rigidity but raise viscosity, whereas low-viscosity diluents such as TEGDMA improve handling and conversion at the expense of greater polymerisation shrinkage and water uptake [8]. The inorganic phase is coupled to this matrix through silane agents, and the integrity of that filler–matrix interface influences both the initial reinforcement and the resistance of the material to hydrolytic breakdown over time [9].

A parallel line of development has been the emergence of universal composites, intended to be bonded reliably with either etch-and-rinse or self-etch adhesive strategies and to serve across a range of cavity classes and shades [10]. Clinicians who select such materials expect high retention, adequate fracture resistance, and a low incidence of postoperative sensitivity, together with esthetic stability under repeated oral challenges [11]. The clinical longevity of posterior composite restorations is now understood to depend on operator factors, patient caries risk, and occlusal load as much as on material chemistry [12].

Filtek™ Z550 (3M ESPE, St. Paul, MN, USA) was introduced in 2012 as a universal nanohybrid and has since become one of the most frequently tested materials of its class, appearing repeatedly as a reference or benchmark in comparative research [4]. Its resin matrix combines Bis-GMA, UDMA, Bis-EMA, TEGDMA, and PEGDMA, and it is reinforced with a high loading of surface-treated zirconia and silica fillers arranged in a bimodal architecture of submicron clusters and discrete nanoparticles [13]. This combination of a well-documented composition, a reproducible microstructure, and an unusually large body of independent testing is the reason it is adopted here as the representative material through which the behaviour of the wider nanohybrid class is examined [14]. Recent clinical work has reported high short-term success for the material in Class II cavities, with an elevated frequency of transient postoperative sensitivity relative to some comparators [15], while laboratory studies have drawn attention to water-induced optical changes and to a measurable loss of surface properties after thermal ageing [16].

The aim of this narrative review was to summarise and discuss the composition, physicomechanical, chemical, optical, and clinically related behaviour of nanohybrid resin composites, examined through Filtek™ Z550 as a representative and extensively studied material, and to place the observed trends in the context of the wider class. Because the review is narrative rather than systematic, no formal quality-appraisal or risk-of-bias assessment was undertaken, and this is acknowledged as a limitation of the synthesis [17].

## 2. Materials and Methods

### 2.1. Review Design

The work was designed as a structured narrative review. It was not registered as a systematic review and did not include a formal risk-of-bias or certainty-of-evidence assessment, so the quality of narrative synthesis was guided by the SANRA framework rather than by a systematic-review protocol [17,18].

### 2.2. Review Question and PICO Framework

The review was organised around the question of how the composition and microstructure of a representative nanohybrid composite relate to its physicomechanical, chemical, optical, and clinical behaviour. The PICO-oriented framework was adapted for a materials-science review as follows: Population or Problem, permanent teeth or laboratory specimens restored with a nanohybrid resin composite; Indicator or Intervention, Filtek™ Z550 evaluated as the tested material; Comparison, other resin composites and restorative materials, or the same material before and after ageing; Outcome, physicomechanical, chemical, optical, or clinical performance measures.

### 2.3. Information Sources and Search Strategy

A comprehensive search was performed in PubMed and adapted for the Cochrane Library using equivalent fields and syntax, and it was supplemented by manual screening of the reference lists of retrieved articles. The searches were conducted in January 2026, and the base strategy combined product terms with material and outcome terms, for example (“Filtek Z550”[tiab] OR “Filtek Z 550”[tiab]) AND (dental OR tooth OR restorative OR composite OR resin). The term “composite” was included alongside “resin” so that shear-bond-strength and ageing studies, which frequently index under “composite,” were captured. PubMed and the Cochrane Library were selected as the primary indexed sources of peer-reviewed dental research; other databases such as Scopus, Embase, and Web of Science were not searched, and only English-language, peer-reviewed publications were considered. These restrictions are acknowledged as a limitation because they may introduce database and language bias.

### 2.4. Eligibility Criteria

The predefined inclusion and exclusion criteria are summarised in Table 1. In brief, studies were eligible when the representative nanohybrid was evaluated explicitly as a tested material, when original experimental or clinical data were reported, and when at least one relevant physicomechanical, chemical, optical, or clinical outcome was assessed. Only English-language, peer-reviewed publications from 2010 to 2025 were included; the lower bound was chosen to bracket the 2012 market introduction of the representative material and to capture its entire period of clinical availability. Conference abstracts, technical reports, dissertations, non-original publications, non-English articles, and grey literature were excluded.

### 2.5. Study Selection

Study selection proceeded in two sequential stages. Titles and abstracts were screened independently by two reviewers (A.-E.C. and I.-I.A.). The full texts of all records retained after this stage were then retrieved and assessed independently by the same two reviewers against the criteria in Table 1, and disagreements or doubtful cases at either stage were resolved by discussion with a senior author (L.-T.C.). The numbers of records identified, screened, retrieved, and excluded at each stage are reported as an outcome of this procedure at the beginning of Section 3.

### 2.6. Data Extraction and Synthesis

Data were extracted into standardised tables developed for this review, capturing study design, specimen preparation, ageing procedure, testing standard, comparator materials, and reported outcomes. Extraction was performed by one reviewer and checked by a second, disagreements were resolved by discussion, and missing data were recorded as not reported rather than imputed. Where possible, units were harmonised to a common convention for each property, and the testing conditions most frequently reported across studies, such as 5000 to 10,000 thermocycles for ageing, were used as reference points for comparison. Given the heterogeneity of designs, protocols, and outcome measures, a quantitative meta-analysis was not appropriate; results were therefore grouped by outcome domain and synthesised qualitatively, with values presented as ranges and interpreted in the context of their testing protocol.

## 3. Results

The searches identified 86 records, 61 from PubMed and 25 from the Cochrane Library. Six duplicates and five records excluded for other predefined reasons were removed, leaving 75 records for title and abstract screening, of which 30 were excluded as irrelevant. Full texts were sought for the remaining 45 reports. Thirteen of these could not be obtained through institutional subscriptions, interlibrary request, or direct contact with the corresponding authors, and were therefore not assessable; this is the reason only 32 of the 45 reports proceeded to full-text assessment. Of those 32 reports, 5 were excluded, 1 as a non-original publication and 4 for insufficient extractable material-specific data, leaving 27 studies for qualitative synthesis. Their characteristics and principal findings, together with the study type and comparator materials, are presented in Table 2.

### 3.1. Material Composition and Microstructure

Across the included literature, Filtek™ Z550 is described consistently as a universal nanohybrid resin-based composite [13]. According to the manufacturer’s technical product profile for this specific product, its organic phase is a dimethacrylate blend of Bis-GMA, UDMA, Bis-EMA, PEGDMA, and TEGDMA, and its inorganic phase combines a non-agglomerated, non-aggregated 20 nm silica filler, a non-agglomerated, non-aggregated 4 to 11 nm zirconia filler, and an aggregated zirconia/silica cluster filler built from those same 20 nm silica and 4 to 11 nm zirconia particles, with a cluster particle size range of 0.6 to 10 µm [42]. The manufacturer reports an inorganic loading of approximately 82 percent by weight, corresponding to roughly 68 percent by volume [42]. A loading as high as 87 percent by weight appears in one secondary source [15]; because that figure cannot be traced to a manufacturer specification or to a stated analytical method, the manufacturer-reported value is adopted throughout this review and the higher figure is recorded as an unresolved discrepancy in the literature rather than explained. The photoinitiator is not specified in the manufacturer’s documentation, and no material-specific determination was found in the included studies; a camphorquinone–amine system is conventional for dimethacrylate composites of this type and is assumed here on that basis only, as noted in Table 3. The principal components are summarised in Table 3; the individual monomer weight fractions are proprietary and are not disclosed, which is a recurring constraint when comparing nanohybrids at the level of matrix chemistry.

The value of this bimodal architecture lies in efficient space filling, since nanoparticles occupy the interstitial regions between larger clusters and raise the attainable filler fraction without compromising the polishable surface [2]. A denser and more homogeneous packing of the inorganic phase is generally associated with higher stiffness and improved wear resistance, provided that each particle is adequately silanated and coupled to the surrounding matrix [9]. The chemistry of that coupling is itself a determinant of performance, because the type and quantity of silane influence both the local degree of conversion near the filler surface and the durability of the interfacial bond under hydrolytic attack [43]. The consistency with which Filtek™ Z550 is adopted as a nanohybrid control in comparative studies reflects this well-defined and reproducible microstructure [14].

### 3.2. Physicomechanical Properties

#### 3.2.1. Flexural Strength and Elastic Modulus

Filtek™ Z550 shows high early strength relative to many experimental and microhybrid formulations, a behaviour that is likely associated with its elevated filler fraction rather than determined by it alone, since particle geometry, silanisation, and curing also contribute [2]. A flexural strength of approximately 120 to 160 MPa in three-point bending is frequently quoted for contemporary nanohybrid composites, but this is a class-level range drawn from a general review published before this material reached the market in 2012, and it is reported here as such rather than as a Z550-specific value [3]. Material-specific flexural data come from a single included study, which tested the material in three-point bending according to ISO 4049 using bar specimens of 2 × 2 × 25 mm [33]. In that study, specimens aged for thirty days in artificial saliva at 37 °C did not reach the 80 MPa minimum that ISO 4049 specifies for this class of restorative, and ten thousand thermal cycles reduced the three-point flexural strength by 16.6 percent, against a reduction of approximately 4 percent for the same material in the biaxial test [33]. This is a material-specific finding of some consequence, because it indicates that a favourable unaged strength profile should not be assumed to persist after hydrothermal ageing. The same study reported fracture loads near 154 N for unaged biaxial specimens; because a force in newtons is not a strength, this value should be read as the recorded fracture load rather than as a flexural strength, and it must be normalised to specimen geometry before comparison with three-point bending data [33]. Flexural modulus rises with filler content, so a higher modulus assists in resisting occlusal deformation while also concentrating stress at the restoration margin and influencing the distribution of polymerisation shrinkage stress [44]. The relationship between filler fraction and mechanical output is not linear, since particle size distribution and the state of filler aggregation modulate the reinforcing efficiency of a given loading [2]. When the material serves as a comparator for industrially processed CAD/CAM composite blocks, its unaged flexural strength remains competitive, although the polymerisation and storage conditions of a chairside material introduce greater variability than the standardised manufacturing of milled blocks [45].

#### 3.2.2. Surface Hardness

Surface hardness is one of the most frequently documented properties of the material, and its Vickers and Knoop values are consistently comparable to or greater than those of contemporary hybrid materials [29]. Reported values depend strongly on the indentation load, dwell time, and storage condition, so results obtained under different protocols cannot be compared directly; for example, a measurement at HV1 cannot be equated with one at HV0.3, and specimens stored dry, in water, or in artificial saliva may differ substantially [46]. With this caveat, Knoop microhardness near 89 and Vickers values close to 86 have been recorded for well-cured, comparably stored specimens [34], and a cavity-depth study reported top-surface Vickers hardness near 111 with a bottom value near 88 [27]. This gradient between the irradiated and deeper layers shows that adequate energy delivery throughout the increment is required to translate a favourable composition into uniform mechanical performance [47]. Because hardness correlates with the degree of conversion, microhardness is often used as an indirect indicator of polymerisation quality, although a high value reflects the filler phase together with the matrix and is best interpreted alongside conversion data [48].

#### 3.2.3. Ageing and Wear Resistance

Evidence from a range of ageing protocols indicates that Filtek™ Z550, in common with other highly filled nanohybrids, is susceptible to degradation under hydrothermal fatigue despite high initial properties [49]. High-cycle thermal simulations of the order of 10,000 cycles consistently reduce surface-layer properties, and microhardness decreases by approximately 16 to 24 percent after prolonged ageing or thermocycling [29]. This softening is likely associated with the plasticising action of absorbed water on the dimethacrylate matrix and with hydrolysis of the silane coupling layer, both of which weaken the filler–matrix interface [34]. Tribological testing confirms that hydrothermal conditioning increases the volumetric wear rate, in some cases approaching a doubling of wear loss relative to unaged specimens [26]. The magnitude of these changes varies with the counter-body, medium, and load, which complicates comparison across studies; the lack of a universally adopted ageing protocol is a recognised source of divergence, and structured proposals for standardising artificial ageing have been advanced specifically to make such comparisons meaningful [50]. Interpreting the ageing literature for any single nanohybrid therefore requires attention to the thermocycling regimen and storage medium as much as to the material itself [51]. Even so, some biaxial fatigue data indicate that bulk strength can be retained after ageing while surface wear resistance declines, which suggests that surface and bulk degradation respond to hydrothermal stress along partly separate pathways [33]. Reported physicomechanical and ageing outcomes are presented in Table 4.

### 3.3. Chemical Properties and Stability

#### 3.3.1. Degree of Conversion and Depth of Cure

The degree of conversion of the material is reported variably according to the curing protocol and specimen depth, with values between roughly 39 and 76 percent [32]. When irradiance and exposure time are adequate, conversion remains high and relatively uniform across a 2 to 4 mm increment, which supports use in deeper posterior cavities when incremental placement is observed [27]. An abbreviated exposure or an insufficient light output lowers conversion and leaves a softer, more soluble polymer, an effect well established for dimethacrylate systems generally [52]. The depth to which adequate conversion extends is governed by the attenuation of light through the increment, so irradiance, exposure time, shade, and translucency jointly determine the effective cure depth [53]. For a conventional nanohybrid such as this material, a layered technique using increments of approximately 2 mm is advisable, because conversion and hardness fall measurably at the base of thicker increments even when the surface appears well polymerised [54]. It should be stated plainly that no included study measured depth of cure for this material by the scraping method specified in ISO 4049, which requires a cured depth of at least 1.5 mm for this class of restorative, and no material-specific depth-of-cure value could therefore be extracted. The degree of conversion and the microhardness measured at depth that are reported above characterise polymerisation quality at a given depth, but they are not equivalent to a depth-of-cure determination and are not offered as a substitute for one; the absence of such a measurement is recorded here as a gap in the available evidence.

#### 3.3.2. Monomer Elution, Cytotoxicity, and Biocompatibility

Incomplete conversion is associated with the release of residual monomers and additives, and studies of Filtek™ Z550 have detected the elution of HEMA, TEGDMA, UDMA, and Bis-GMA in aqueous or ethanolic media, with HEMA typically the most abundant and TEGDMA among the lowest by high-performance liquid chromatography [36]. Direct chromatographic quantification specific to this material remains limited, and much of the available evidence is comparative or qualitative rather than a full elution profile [55]. Reported eluate concentrations generally remain below established toxic thresholds and decline over time as the readily extractable fraction is depleted, and even after bleaching, the released amounts have been described as biologically acceptable [36]. In vitro work nonetheless establishes a positive relationship between a lower degree of conversion and greater cytotoxicity of the eluates, with reduced fibroblast metabolic activity for poorly cured specimens [32]. Biocompatibility, however, is broader than cytotoxicity and also encompasses irritation, sensitisation, and genotoxic potential; the cytotoxic and genotoxic effects of dimethacrylate monomers are mediated in part by oxidative stress and disruption of cellular redox balance, mechanisms characterised in detail for TEGDMA and HEMA [56]. Extending the polymerisation time raises fibroblast viability for this material, which links curing discipline directly to biocompatibility [23].

#### 3.3.3. Water Sorption and Solubility

Water sorption is a defining factor in the long-term chemical stability of nanohybrid composites [8]. Filtek™ Z550 shows measurable sorption, and a value of approximately 54 µg/mm^3^ has been recorded after thirty days of immersion in distilled water [16]. This exceeds the 40 µg/mm^3^ sorption limit of ISO 4049, but the comparison is indicative rather than a compliance judgment, because ISO 4049 specifies a seven-day immersion at 37 °C after conditioning to constant mass, whereas the reported value was obtained over thirty days; the limits of applying the ISO protocol outside its specified conditions have been examined directly [57]. In the same study, water solubility for this material was negative, that is, the specimens showed a net mass gain rather than a measurable loss, which the authors attributed to water retention during extended immersion [16]. A negative result of this kind cannot be compared against the 7.5 µg/mm^3^ ISO 4049 solubility limit, so no solubility compliance judgment is made here; a seven-day determination performed according to the standard would be required, and none was found for this material. The absorbed water plasticises the polymer, contributes to the microhardness reduction observed after ageing, and can promote hydrolytic scission of ester bonds and of the silane interface [7]. Hygroscopic expansion also modulates internal stress, since it can partially relax residual polymerisation contraction stress while simultaneously plasticising the network [58]. Compared with industrially processed CAD/CAM materials, a directly cured composite of this type shows greater sensitivity to hydration and re-desiccation, which is reflected in measurable increases in surface roughness and in internal structural change [16].

### 3.4. Optical and Esthetic Properties

#### 3.4.1. Translucency and Gloss

The optical performance of nanohybrid composites is shaped by the size of their filler particles, which, being small relative to the wavelengths of visible light, limit scattering and preserve translucency [59]. For Filtek™ Z550, translucency and opalescence remain comparatively stable during environmental challenge when set against microhybrid comparators, consistent with the influence of matrix composition and filler refractive matching on light transmission [16]. Initial surface gloss is rated highly, with baseline values near 70 gloss units, reflecting the fine finish attainable on a nanohybrid surface [37]. This gloss is not durable under chemical attack, however, and thirty days of exposure to acidic beverages such as cola reduces gloss substantially, toward roughly 38 to 50 gloss units, with comparable losses in distilled-water controls [37]. The finishing and polishing system influences both the initial gloss and its retention, so that material selection and finishing protocol act together to determine esthetic durability [60]. Comparative work confirms that the choice of polishing system alters the final roughness and gloss of nanohybrid surfaces to a clinically meaningful degree, and that toothbrushing simulation further reduces gloss over time [61].

#### 3.4.2. Colour Stability

Colour stability depends strongly on the type and duration of the staining or ageing challenge [62]. Filtek™ Z550 resists intrinsic discolouration better than several flowable and single-shade comparators, and it has recorded low colour change after exposure to zinc-containing mouthrinses and to inhaled asthma medications [38]. Under more aggressive conditions, the material discolours perceptibly, and colour differences frequently exceed the clinically accepted threshold after conventional or laser-activated bleaching and after immersion in low-pH dietary supplements followed by coffee staining [30]. These values must be read against the threshold set that corresponds to the colour-difference formula actually used, because the CIELAB and CIEDE2000 formulas are not interchangeable: the 50:50 perceptibility and acceptability thresholds are approximately 1.2 and 2.7 units for ΔEab, and approximately 0.8 and 1.8 units for ΔE00 [63]. The two formulas weight lightness, chroma, and hue differently, so the same pair of measurements yields different numerical differences and cannot be judged against a common limit [64]. Every colour result summarised in this review was reported as a CIELAB colour difference where the source stated its formula, and several of the included studies applied an older acceptability value of 3.3 ΔEab units rather than the 2.7 threshold derived from more recent threshold research [16,38]. Table 5 therefore identifies the metric for each colour result, records explicitly where a source did not state the formula it used, and applies the matching threshold in each case; a single acceptability value has not been applied across different formulas. The consistent message is that esthetic success is tied to the chemical nature of the external challenge and to patient dietary and treatment factors rather than to an intrinsic instability of the material [65]. Set against newer single-shade and colour-adjusting composites, the multi-shade formulation has shown competitive or superior short-term colour retention and provides a stable reference for evaluating the colour-adaptation claims of these newer systems [66]. Reported chemical, biological, optical, and colour outcomes are summarised in Table 5.

### 3.5. Adhesion, Bonding, and Clinical Outcomes

#### 3.5.1. Bond Strength and Durability

Because nanohybrid composites are frequently placed with universal adhesives, the bond behaviour of Filtek™ Z550 has been examined across several substrates and conditions [10]. Shear bond strength to sound dentine near 22 MPa has been reported for non-diabetic controls, and a self-etch approach improved bonding to diabetic dentine relative to an etch-and-rinse approach, which indicates that substrate condition interacts with adhesive strategy [20]. Several of these bond studies incorporated artificial ageing, typically 1000 to 10,000 thermocycles, and the reported values are therefore post-ageing rather than immediate; the repair bond strength of 27.4 MPa, for instance, was recorded after 10,000 brushing and 10,000 thermal cycles, and the calcium–silicate bonding data were obtained after 10,000 thermocycles [41]. Dedicated long-term durability data that track the decline of the same bond over multiple ageing intervals remain scarce, and this gap is noted rather than filled by the current evidence [40]. Contamination of the bonding surface by hemostatic agents reduced shear bond strength, with iron sulfate more detrimental than aluminium chloride [39]. Over calcium–silicate substrates, bond strength increased with a delay before restoration, so that a short waiting period over one tricalcium–silicate cement and a longer interval over another produced clinically acceptable values [40]. The material also performs well in repair, achieving high repair bond strength when the surface is roughened and silanated, with air abrasion required for durable adhesion to aged resin nanoceramic substrates [35].

#### 3.5.2. Handling and Clinical Performance

Qualitative reports describe the material as having favourable handling, with adequate sculptability and ease of placement using incremental techniques [14]. Clinical evidence is drawn mainly from short-term trials of Class II restorations placed with universal adhesives [15]. Over an eighteen-month follow-up, restorations demonstrated high retention with no recorded failures, although a higher incidence of transient postoperative sensitivity at one week was observed relative to a microhybrid comparator, a finding attributed to polymerisation shrinkage stress in Bis-GMA-based matrices and to the behaviour of the adhesive interface [15]. Postoperative sensitivity in composite restorations is multifactorial and is influenced by adhesive strategy and technique in addition to material stiffness [67]. The available data support predictable short-term performance, but evidence on long-term clinical wear in stress-bearing areas remains limited, so these clinical inferences are preliminary [12].

## 4. Discussion

The synthesised evidence indicates that the behaviour of a highly filled nanohybrid such as Filtek™ Z550 is governed principally by the interaction between its inorganic filler loading and the bimodal architecture of zirconia and silica clusters combined with discrete silica nanoparticles [13]. This configuration is associated with high early mechanical strength and favourable optical properties, and it underpins the use of such materials in both anterior and posterior situations [2]. The dense packing of nanometric fillers supports the flexural strength commonly reported for the class while enabling the fine, polishable surface required for esthetics [5]. The frequent adoption of Filtek™ Z550 as a nanohybrid reference is a practical consequence of this reproducible relationship between structure and property [14].

Placing the representative material against named comparators clarifies where it sits within the class. In simulated parafunctional loading, it retained anatomical form and showed higher initial microhardness than the nanohybrid Evetric [13]. Its gloss at baseline exceeded that of Evetric and remained competitive with GrandioSO and an ormocer comparator, although all materials lost gloss under acidic immersion [37]. Against the microhybrid Filtek Z250, it maintained a smoother surface after combined gastric-acid and toothbrushing challenge [31]. In a separate study using a different challenge, it showed higher colour stability than the bulk-fill composite SonicFill after immersion in mouthrinses [22]. In colour-stability testing against single-shade materials, it outperformed several single-shade and flowable comparators over the short term [25]. These comparisons position the material near the upper range of the nanohybrid class for early hardness and gloss, while confirming that its susceptibility to hydrolytic and acidic degradation is shared with its peers rather than unique to it [2].

A consistent counterpoint to these advantages is the susceptibility of the class to hydrothermal fatigue [49]. Repeated thermal cycling reduces surface microhardness and increases volumetric wear, and both effects point to a progressive compromise of the filler–matrix interface [26]. The most plausible mechanism combines water-induced plasticisation of the dimethacrylate network with hydrolytic degradation of the silane coupling layer [7]. Esterase-mediated hydrolysis of ester bonds in saliva provides an additional degradation pathway that water-storage models capture only partially [68]. The observation that bulk flexural strength can remain high after ageing while surface wear resistance declines suggests that surface and bulk degradation should be evaluated as distinct phenomena [33]. Because the wear and hardness literature is sensitive to the ageing protocol, the recent effort to standardise artificial ageing is directly relevant to interpreting between-study differences for any nanohybrid [50].

The chemical behaviour of the material reinforces the central role of the curing protocol, which the evidence suggests is at least as important as hydrolytic degradation in determining outcome. Monomer elution decreases over time and generally remains below toxic thresholds, yet the documented association between reduced conversion and increased in vitro cytotoxicity means that inadequate light delivery can turn a biocompatible material into a source of cellular stress [32]. This concern is mechanistically grounded, because residual TEGDMA and HEMA disturb cellular redox balance and can trigger oxidative DNA damage, so maximising conversion is a direct route to improved biocompatibility [69]. The demonstrated benefit of extended polymerisation time for fibroblast viability gives this principle a concrete clinical form [23]. Ensuring high conversion also improves mechanical maturity and reduces water sorption, which links the biological and mechanical arguments for disciplined curing [70].

Beyond bulk degradation, the marginal interface remains the principal site of long-term failure for composite restorations, where hydrolytic and enzymatic breakdown can widen the gap and facilitate bacterial ingress [71]. Secondary caries and fracture are consistently the leading reasons for the replacement of posterior composites, so the susceptibility of a highly filled direct material to interfacial degradation carries clinical weight [72]. This reinforces the view that the durability of a restoration is a property of the whole assembly rather than of the bulk material in isolation [11].

The optical findings display a comparable protocol dependence. The material maintains high initial gloss and stable translucency relative to microhybrid comparators [59], yet under aggressive chemical conditions gloss falls sharply and colour change exceeds the acceptability threshold, particularly after bleaching and low-pH exposure [28]. Because these thresholds are well defined in the colorimetric literature, the clinical relevance of the reported changes can be judged with reasonable confidence, and the practical implication is that patient counselling on staining agents and the selection of an appropriate finishing system are integral to esthetic longevity [64]. The finishing and polishing protocol deserves particular emphasis, since surface roughness and gloss retention depend on the abrasive system to a degree comparable with the influence of material choice itself [73].

The reference role of the material is increasingly tested against milled and additively manufactured restoratives [74]. Milled resin-composite blocks generally display more consistent flexural behaviour and lower staining susceptibility than direct composites owing to their industrial polymerisation [75], whereas additively manufactured resins remain sensitive to post-processing variables such as washing regimen and build orientation [76]. Bonding a direct nanohybrid to these substrates during repair depends on appropriate surface conditioning, which frames the material as a repair medium as well as a standalone restorative in the contemporary digital workflow [77].

The clinical evidence, although limited to short-term evaluation, is consistent with adequate retention and fracture resistance in posterior stress-bearing areas [15]. Reports of transient postoperative sensitivity indicate that polymerisation shrinkage stress remains a relevant concern in Bis-GMA-based systems, which reinforces the value of incremental placement, controlled curing, and well-chosen adhesives [44]. Set against the wider evidence on posterior composite longevity, in which patient risk profile and operator technique dominate outcome, the material can be regarded as a dependable representative of its class in the laboratory, while its clinical longevity remains to be established by longer trials [12].

## 5. Limitations

Several limitations constrain the strength of the conclusions. First, the synthesis is narrative rather than systematic: no formal risk-of-bias, quality-appraisal, or certainty-of-evidence assessment was performed, and narrative reviews are inherently more prone to selection bias than systematic reviews [17]. Second, the search was limited to PubMed and the Cochrane Library and to English-language, peer-reviewed publications, with no interrogation of Scopus, Embase, or Web of Science and no inclusion of grey literature; these restrictions may introduce database and language bias and may have excluded relevant evidence [18]. Third, thirteen of the forty-five reports sought in full text could not be obtained, so the synthesis rests on the 32 reports that were assessable. Fourth, there is substantial methodological heterogeneity across the included studies, since thermocycling parameters, wear counter-bodies, curing irradiances, colour-difference formulas, and staining media differ from one report to another, which precludes formal pooling [50]. It is important to separate the two evidence streams that the synthesis combines. The in vitro stream, which supplies 26 of the 27 included studies, cannot reproduce continuous salivary enzyme activity, the mechanical complexity of mastication, or the influence of oral biofilm, so laboratory outcomes may under- or over-estimate clinical behaviour [51]. The clinical stream consists of a single included trial with an eighteen-month follow-up, and the absence of any longer randomised controlled trial prevents a definitive assessment of fatigue resistance and failure patterns over a clinically meaningful lifespan [15].

## 6. Conclusions

Within the limits of a narrative synthesis, and restricted to the evidence reviewed for this material, the laboratory data indicate that Filtek™ Z550 combines a reproducible bimodal filler architecture with high early mechanical strength, elevated surface hardness, and favourable polishability. The same in vitro evidence identifies a reproducible vulnerability to hydrothermal fatigue, expressed as a progressive reduction in surface integrity and increased wear after thermal ageing, together with an esthetic stability that is high initially but declines under aggressive chemical challenge. In the single study that tested the material in three-point bending according to ISO 4049, flexural strength fell below the 80 MPa minimum after thirty days of ageing in artificial saliva, so the favourable unaged profile should not be assumed to persist in service. Polymerisation quality emerges as a determinant of outcome comparable in importance to hydrolytic degradation, because conversion governs mechanical maturity, water sorption, and biocompatibility simultaneously. These statements describe Filtek™ Z550 as characterised by the 27 studies reviewed here and are not extended to nanohybrid composites as a class, since the comparative data assembled for this review are insufficient to support a class-level generalisation. They rest on laboratory data and should be distinguished from the clinical evidence, which comprises a single eighteen-month trial reporting high retention accompanied by a higher incidence of transient postoperative sensitivity; statements about clinical suitability are therefore preliminary. Future work should prioritise standardised ageing and wear protocols, depth-of-cure and solubility determinations performed according to ISO 4049, direct comparison with milled and additively manufactured materials under matched conditions, and long-term randomised clinical trials, so that the laboratory profile summarised here can be translated into reliable estimates of clinical service life.

## Figures and Tables

**Table 1:** Predefined inclusion and exclusion criteria applied during study selection.

Inclusion Criteria	Exclusion Criteria
Filtek™ Z550 evaluated explicitly as a tested restorative material	Filtek™ Z550 not investigated as a tested material
Original in vitro (laboratory) or clinical data reported	Non-original publications (reviews, editorials, letters, opinion pieces)
At least one relevant physicomechanical, chemical, optical, or clinical outcome assessed	Insufficient extractable or material-specific outcome data
Peer-reviewed, English-language article published between 2010 and 2025	Unrelated dental applications without material-specific outcomes
Sufficient methodological detail for interpretation and qualitative comparison	Conference abstracts, dissertations, technical reports, and other grey literature; non-English publications

**Table 2:** Characteristics and principal findings of the 27 included studies, with study type and comparator materials. All studies evaluated Filtek™ Z550 as a tested or reference material.

Study (Author, Year)	Type	Focus/Design	Main Comparators	Principal Finding for Filtek™ Z550
Candan et al., 2023 [19]	In vitro	SBS over calcium–silicate substrates	EverX Flow/Posterior	Highest SBS when restoration delayed 7 days (25.8–39.3 MPa)
Attia et al., 2024 [20]	In vitro	SBS to dentine in diabetes; adhesive mode	Non-diabetic vs. Type 1/2 diabetic	≈21.7 MPa in controls; diabetes lowers bond >30%; self-etch aids diabetic dentine
Pala et al., 2016 [21]	In vitro	Hardness, roughness, gloss, colour after polishing and thermocycling	Majesty Posterior, G-aenial	Lowest ΔE with Enhance system; hardness falls after 10,000 cycles
Toz-Akalın et al., 2016 [22]	In vitro	Colour stability against mouthrinses	SonicFill	Higher colour stability than SonicFill; ΔE 2.25 with alcohol rinse
Atalayın Özkaya et al., 2021 [23]	In vitro	Extended curing on ROS and cell viability	Admira Fusion, Ceram.X, Solare X	40 s curing raises fibroblast viability (35.3% → 69%)
Popescu et al., 2023 [24]	In vitro	Wear after bleaching protocols	G-aenial, Gradia, Valux Plus	Office 40% bleaching raises wear (trace 275.7 µm)
Özden & Karadaş, 2025 [15]	Clinical	Class II restorations, 18 months	G-aenial Posterior	100% retention and fracture resistance; 9.5% postoperative sensitivity
Tepe et al., 2025 [25]	In vitro	Colour stability of single-shade composites	Omnichroma, Vittra, Charisma One	Lowest short-term ΔE1 (2.85); ΔE2 (5.87) exceeds threshold
Boitor et al., 2023 [13]	In vitro	Simulated bruxism and ageing (CBCT/X-ray)	Evetric	Smoother post-ageing (Grade 0); interfacial defects after 70 N
Pieniak et al., 2019a [26]	In vitro	Thermocycling on surface layer, sliding pair	Filtek Flow, experimental	Vickers 67.9 → 48.5 after 10,000 cycles
Gömleksiz & Gömleksiz, 2025 [27]	In vitro	Cavity depth on DC and microhardness	Clearfil Majesty, Beautifil II LS	DC ~76% at 2 and 4 mm; Vickers top 111 / bottom 88
Osiceanu et al., 2025 [16]	In vitro	Water sorption on surface and colour, direct vs. CAD/CAM	Gradia, Tetric Evo, Vita Enamic	Water sorption 53.9 µg/mm^3^; optical fluctuation; Ra < 0.2 µm
Mawlood & Hamasaeed, 2023 [28]	In vitro	Diode-laser 940 nm bleaching on colour	Filtek Bulk Fill, Z350 XT Flow	ΔE 9.50 (conventional)/8.90 (laser); both unacceptable
Pieniak et al., 2023 [29]	In vitro	Hydrothermal factors on microhardness	Filtek Bulk-Fill	Knoop ~89 initial; 22–24% hardness loss after cycling
Tzimas et al., 2025 [14]	In vitro	Surface characteristics, subtractive vs. additive	Shofu HC, Vita Enamic, VarseoSmile	82 wt%; high polishability (Sa ~57 nm); benchmark control
Çınar et al., 2023 [30]	In vitro	Low-pH dietary supplements on discolouration	Filtek One Bulk-Fill, Fill-Up	ΔE 3.59 (supplement) to 15.11 (after coffee)
Tărăboanță et al., 2022 [31]	In vitro	Gastric acid and toothbrushing on surface	Filtek Z250, Herculite XRV	Ra 0.222 → 0.376 µm; better than Z250 microhybrid
Małkiewicz et al., 2017 [32]	In vitro	Incomplete polymerisation and cytotoxicity	Gradia, Herculite, Tetric, Charisma	DC 39.3%; 3T3 activity 81.5%; cytotoxic potential
Pieniak et al., 2019b [33]	In vitro	Biaxial flexural hydrothermal fatigue	Experimental nano/flow	Fracture load 154 N; ~4% strength loss; modulus rises
Pieniak et al., 2020 [34]	In vitro	Hardness and wear, humid non-stationary temperatures	Experimental nano/flow	Vickers 85.9 → 81.2; wear 1.68 × 10^8^ µm^3^; highest hardness
Subaşı & Alp, 2017 [35]	In vitro	Repair bond strength of aged/non-aged nanoceramics	Lava Ultimate	Air abrasion needed; aged untreated ~9.1 → 13.0 MPa
Gül et al., 2019 [36]	In vitro	Monomer release after bleaching (HPLC)	Tetric N-Ceram	HEMA highest, TEGDMA lowest; below toxic doses ≥28 days
Zovko et al., 2023 [37]	In vitro	Chemical degradation and polishing on gloss	Admira Fusion, GrandioSO, Evetric	Baseline 70.5 GU; ~44% gloss loss; higher than Evetric
Candan & Ünal, 2024 [38]	In vitro	Inhaled asthma medications on colour (paediatric)	Fuji II LC, Dyract XP, Filtek Ultimate	Lowest ΔE (1.09–1.77); most recommended material
Khamverdi et al., 2021[39]	In vitro	Hemostatic contamination on SBS	G-Premio vs. Single Bond Universal	AlCl_3_ and Fe_2_(SO_4_)_3_ lower SBS; Fe_2_(SO_4_)_3_ more harmful
Çeliksöz & Irmak, 2024 [40]	In vitro	Delayed vs. immediate placement over Biodentine/RetroMTA	EQUIA Forte, Theracal LC	µSBS 1.84 → 6.37 MPa over Biodentine; 12-min wait sufficient
Ozaslan et al., 2023 [41]	In vitro	Repair bond strength vs. self-adhesive materials	Cention-N, EQUIA Forte	Highest repair bond (27.4 MPa); superior for repair

Abbreviations: CBCT, cone–beam computed tomography; DC, degree of conversion; GU, gloss units; HPLC, high-performance liquid chromatography; Ra, arithmetical mean profile roughness; ROS, reactive oxygen species; Sa, arithmetical mean height of the surface (areal roughness); SBS, shear bond strength; µSBS, micro-shear bond strength; ΔE, colour difference; wt%, percent by weight. Comparator materials and their manufacturers: Admira Fusion, GrandioSO (VOCO, Cuxhaven, Germany); Beautifil II LS, Shofu Block HC (Shofu, Kyoto, Japan); Biodentine (Septodont, Saint-Maur-des-Fossés, France); Cention N, Evetric, Tetric Evo, Tetric N-Ceram (Ivoclar Vivadent, Schaan, Liechtenstein); Ceram.X, Dyract XP (Dentsply Sirona, Charlotte, NC, USA); Charisma, Charisma One (Kulzer, Hanau, Germany); Clearfil Majesty Posterior (Kuraray Noritake, Tokyo, Japan); EQUIA Forte, EverX Flow, EverX Posterior, Fuji II LC, G-aenial, G-aenial Posterior, Gradia, G-Premio Bond, Solare X (GC, Tokyo, Japan); Fill-Up (Coltene, Altstätten, Switzerland); Filtek Bulk Fill, Filtek Flow, Filtek One Bulk-Fill, Filtek Ultimate, Filtek Z250, Filtek Z350 XT Flow, Lava Ultimate, Single Bond Universal, Valux Plus (3M, St. Paul, MN, USA); Herculite XRV, SonicFill (Kerr, Brea, CA, USA); Omnichroma (Tokuyama Dental, Tokyo, Japan); RetroMTA (BioMTA, Seoul, Republic of Korea); TheraCal LC (BISCO, Schaumburg, IL, USA); VarseoSmile Crown Plus (BEGO, Bremen, Germany); Vita Enamic (VITA Zahnfabrik, Bad Säckingen, Germany); Vittra APS (FGM, Joinville, Brazil).

**Table 3:** Reported composition of the representative nanohybrid composite (Filtek™ Z550), compiled from manufacturer information and the included studies.

Component	Description	Reported Amount
Resin matrix	Bis-GMA, UDMA, Bis-EMA, PEGDMA, TEGDMA (dimethacrylate blend) [42]	Individual monomer fractions proprietary; not disclosed
Photoinitiator system	Camphorquinone with tertiary-amine co-initiator—assumed; not specified by the manufacturer and not determined in any included study	Not disclosed
Nanoparticle filler	Non-agglomerated, non-aggregated silica (20 nm) and zirconia (4–11 nm) [42]	Part of total inorganic load
Cluster (base) filler	Aggregated zirconia/silica clusters formed from the same 20 nm silica and 4–11 nm zirconia particles; cluster size 0.6–10 µm [42]	Part of total inorganic load
Total inorganic filler	Combined nanoparticle and cluster system [42]	≈82 wt% (≈68 vol%)
Indications/shades	Universal; anterior and posterior direct restorations [42]	Multiple VITA-based shades

Bis-GMA, bisphenol-A glycidyl dimethacrylate; UDMA, urethane dimethacrylate; Bis-EMA, bisphenol-A ethoxylated dimethacrylate; PEGDMA, poly(ethylene glycol) dimethacrylate; TEGDMA, triethylene glycol dimethacrylate. Filler types, particle sizes, and loading are those specified by the manufacturer for Filtek™ Z550 [42]. The photoinitiator is not specified by the manufacturer and was not determined in any included study; the entry given is an assumption based on the conventional chemistry of this material class and should be read as such. Exact monomer weight fractions are not released by the manufacturer.

**Table 4:** Reported physicomechanical and hydrothermal-ageing outcomes for Filtek™ Z550, with alignment to ISO 4049 where applicable.

Property/Metric	Reported Value(s)	Test Condition	ISO/Reference Alignment	Ref.
Flexural strength (class-level range, not Z550-specific)	~120–160 MPa	Three-point bending, unaged; general nanohybrid range	Quoted against the ISO 4049 minimum of 80 MPa; source predates the 2012 market introduction of Z550	[3]
Flexural strength (Z550-specific)	Below the 80 MPa ISO minimum after ageing; −16.6% after thermocycling	Three-point bending per ISO 4049, 2 × 2 × 25 mm bars; 30 d artificial saliva at 37 °C; 10,000 thermal cycles	Does not meet ISO 4049 (≥80 MPa) after ageing	[33]
Biaxial fracture load	154 N (control)	Piston-on-three-ball, before ageing	Not an ISO strength value	[33]
Biaxial strength loss	~4% (lowest observed)	After 10,000 thermal cycles	Not an ISO parameter	[33]
Vickers microhardness	85.9 → 81.2	0 → 10,000 thermal cycles (artificial saliva)	No ISO limit	[34]
Knoop microhardness	~89 (initial)	Well-cured, higher than bulk-fill comparator	No ISO limit	[29]
Microhardness loss	22–24%	After thermocycling	No ISO limit	[29]
Vickers hardness (depth)	Top 111/bottom 88	2–4 mm increments, 1000 mW/cm^2^, 20 s	No ISO limit; not a depth-of-cure determination	[27]
Vickers microhardness (PMCC)	67.9 → 48.5	0 → 10,000 thermal cycles, sliding pair	No ISO limit	[26]
Wear volume/friction work	1.68 × 10^8^ µm^3^/~870 J	Ball-on-disc, 10,000 thermal cycles	No ISO limit	[34]
Wear trace width	275.7 µm (office bleach)	20,000 friction cycles after bleaching	No ISO limit	[24]
Surface roughness (Ra)	0.222 → 0.376 µm	Gastric acid + toothbrushing	Above the 0.2 µm plaque-retention threshold already at baseline, and further after challenge	[31]
Repair bond strength	27.4 ± 3.7 MPa	Bur + silane + universal adhesive, after 10,000 brushing and 10,000 thermal cycles	No ISO limit; highest of the materials compared in the source study	[41]

**Table 5:** Reported chemical, biological, optical, and colour outcomes for Filtek™ Z550, with alignment to reference thresholds where applicable.

Property/Metric	Reported Value(s)	Test Condition	ISO/Reference Alignment	Ref.
Degree of conversion	~76% (at 2 and 4 mm)	FTIR; 1000 mW/cm^2^, 20 s per 2 mm layer	Adequate (>55% target)	[27]
Degree of conversion	39.3% (at 1 mm)	20 s, 1100 mW/cm^2^	Below adequacy target	[32]
Depth of cure	Not reported	ISO 4049 scraping method not performed in any included study	ISO 4049 requires ≥1.5 mm; not assessable from the available evidence	—
Fibroblast viability	35.3% → 69% (72 h)	20 s vs. 40 s LED curing	ISO 10993-5 context	[23]
Monomer release	HEMA highest, TEGDMA lowest	HPLC, ≥28 days, after bleaching	Below reported toxic doses	[36]
Water sorption	53.9 µg/mm^3^	30-day immersion, distilled water	Above the ISO 4049 limit of 40 µg/mm^3^, but ISO specifies 7-day immersion; indicative only, not a compliance judgment	[16]
Water solubility	Negative (net mass gain; water retention)	30-day immersion, distilled water	Cannot be compared with the ISO 4049 limit of 7.5 µg/mm^3^; no 7-day ISO determination available	[16]
Baseline gloss	70.5 GU; ~44% loss	30-day immersion (cola/tea/water)	No ISO limit	[37]
Colour change (bleaching)	ΔEab 9.50/8.90	Conventional vs. 940 nm laser; formula not stated in source	Exceeds the ΔEab acceptability threshold of 2.7	[28]
Colour change (ageing)	ΔEab1 2.85/ΔEab2 5.87	Brushing + coffee + thermocycling; formula not stated in source	ΔEab2 exceeds the ΔEab acceptability threshold of 2.7	[25]
Colour change (asthma meds)	ΔEab 1.09–1.77	21-day nebulisation; CIELAB stated in source	Within the ΔEab acceptability threshold of 2.7	[38]
Colour change (low-pH supp.)	ΔEab 3.59 → 15.11	28-day supplement then coffee; formula not stated in source	Exceeds the ΔEab acceptability threshold of 2.7	[30]
Colour change (thermocycling)	ΔEab 1.28–2.25	10,000 cycles, polishing systems; formula not stated in source	Below the ΔEab acceptability threshold of 2.7	[21]

## Data Availability

The original contributions presented in the study are included in the article; further inquiries can be directed to the corresponding authors.
